# Keap1-Nrf2 Heterodimer: A Therapeutic Target to Ameliorate Sickle Cell Disease

**DOI:** 10.3390/antiox12030740

**Published:** 2023-03-17

**Authors:** Waseem Chauhan, Rahima Zennadi

**Affiliations:** Division of Hematology, Duke Comprehensive Sickle Cell Center, Department of Medicine, Duke University School of Medicine, Research Drive, Durham, NC 27710, USA

**Keywords:** sickle cell disease, Nrf2-Keap1 signaling, gamma globin, oxidative stress, antioxidant response element, ROS, therapeutic drugs, cytoprotection

## Abstract

Sickle cell disease (SCD) is a monogenic inheritable disease characterized by severe anemia, increased hemolysis, and recurrent, painful vaso-occlusive crises due to the polymerization of hemoglobin S (HbS)-generated oxidative stress. Up until now, only four drugs are approved for SCD in the US. However, each of these drugs affects only a limited array of SCD pathologies. Importantly, curative therapies, such as gene therapy, or hematopoietic stem cell transplantation are not available for every patient because of their high costs, availability of donor matching, and their serious adverse effects. Therefore, there is an unmet medical need for novel therapeutic strategies that target broader SCD sequelae. SCD phenotypic severity can be alleviated by increasing fetal hemoglobin (HbF) expression. This results in the inhibition of HbS polymerization and thus sickling, and a reduction in oxidative stress. The efficacy of HbF is due to its ability to dilute HbS levels below the threshold required for polymerization and to influence HbS polymer stability in RBCs. Nuclear factor-E2-related factor 2 (Nrf2)/Kelch-like ECH-associated protein-1 (Keap1)-complex signaling is one of the most important cytoprotective signaling controlling oxidative stress. Nrf2 is present in most organs and, after dissociation from Keap1, it accumulates in the cytoplasm, then translocates to the nucleus where it binds to the antioxidant response element (ARE) sequences and increases the expression of various cytoprotective antioxidant genes. Keeping this in mind, various researchers have proposed a role of multiple agents, more importantly tert-Butylhydroquinone (tBHQ), curcumin, etc., (having electrophilic properties) in inhibiting keap1 activity, so that Nrf2 can translocate to the nucleus to activate the gamma globin gene, thus maintaining alpha-hemoglobin-stabilizing protein (AHSP) and HbF levels. This leads to reduced oxidative stress, consequently minimizing SCD-associated complications. In this review, we will discuss the role of the Keap-1–Nrf2 complex in hemoglobinopathies, especially in SCD, and how this complex might represent a better target for more effective treatment options.

## 1. Introduction

Sickle cell disease (SCD) and β-thalassemia are the two major β-globin-associated recessively inherited hemoglobinopathies in which mutation in the β-globin gene causes reduced synthesis or improper functioning of hemoglobin due to an imbalance between the α- and β-chain [1]. In SCD, a single base is changed from adenine (A) to thymine (T) in the β-globin gene which leads to the replacement of glutamate by valine at the sixth position in the β-globin chain and the production of sickle hemoglobin (HbS). HbS has a lesser binding affinity towards oxygen than HbA (normal Hb); this reduced oxygen affinity causes polymerization of HbS, declining a greater oxygen binding affinity. Irrespective of oxygen partial pressure, the reduced affinity of HbS towards oxygen increases the tense conformation or T state during which hemoglobin is deoxygenated; a state furthering polymerization of HbS, which causes sickling of RBCs [2]. In SCD, oxidative stress is due to cyclic polymerization/depolymerization of HbS. Various reports have suggested that in SCD, the balanced redox reaction is disturbed due to catalysis of the Fenton reaction by the free hemoglobin released as a result of intravascular hemolysis, the recurrent ischemia-reperfusion injury promoting activation of the xanthine-xanthine oxidase system, and higher autoxidation of HbS generating reactive oxygen species (ROS) [3,4]. Protein phosphorylation, the activation of various transcription factors, apoptosis, immunity, and cell differentiation are all processes that depend on the proper generation and presence of ROS inside the cell, which must be controlled at low levels. As ROS production rises, it negatively impacts crucial cellular components such as proteins, lipids, and nucleic acids, ensuing oxidative stress [5,6]. The cellular antioxidant defense system is compromised in the pathogenesis of not only SCD, but in many other diseases as well, including Alzheimer’s, Parkinson’s, diabetes, atherosclerosis, metabolic disorders, cardiovascular diseases, and cancer [7,8]. As a result, accumulated ROS remains non-neutralized, causing oxidative burden, which contributes to SCD-associated complications and, more importantly, severe anemia and recurrent painful vaso-occlusive crises (VOC), the hallmarks of the disease [3,9,10].

To maintain redox homeostasis of the cell, ROS are counterbalanced by intricate numerous antioxidant systems. The nuclear factor-E2-related factor 2 (Nrf2)/Kelch-like ECH-associated protein-1 (Keap1) signaling is the most prominent antioxidant system. A genome-wide chromatin immunoprecipitation (ChIP)-seq has suggested that the Nrf2/Keap1–antioxidant response element (ARE) signaling pathway is highly sensitive to oxidative stress, in which Nrf2 promotes the transcription of multiple antioxidant genes by binding to AREs in the nucleus [11,12]. In this review, we will discuss the basic roles of Nrf2 and how Nrf2 is now thought to be the prime target to treat hemoglobinopathies, especially SCD.

## 2. Oxidative Stress in SCD

SCD is a complicated pathophysiologic disorder caused in part by several pro-oxidant mechanisms, resulting in chronic and systemic oxidative stress. Erythrocytes are continuously exposed to a free radical environment in healthy biological systems [8]. However, potential indicators of the severity of SCD include increased ROS generation and the byproducts of their oxidative reactions [13]. In SCD patients, the main source of pro-oxidants is the sickle erythrocyte, where unstable autoxidative HbS and higher metabolic turnover due to recurrent HbS polymerization and depolymerization produce enhanced ROS formation. In addition, sickle RBCs are subjected to continuous exogenous oxidative onslaughts, contributing to the evolution of SCD vasculopathy. Notably, nicotinamide adenine dinucleotide phosphate oxidases (NADPH oxidases), reported in sickle RBCs, may act as an incubator for oxidized Hb redox forms [14,15,16]. Further, ROS accumulation damages key sickle cell components. Increased ROS generation in sickle RBCs could contribute to cell membrane damage and premature hemolysis [17], and trigger sickle cell adhesion [15,18], consequently leading to severe anemia, vaso-occlusion, increased susceptibility to infections, chronic inflammatory diseases, and microvascular damage in organs [19]. These multifactorial events create a cyclic cascade, resulting in higher levels of ROS and oxidative damage, lower quality of life and life expectancy [20,21]. Many investigations have found that sickle erythrocytes produce twice as many superoxide (O_2_^•−^), hydrogen peroxide (H_2_O_2_), hydroxyl radical (HO^•^), and lipid oxidation products as HbA-containing erythrocytes [22]. The two primary antioxidant enzymes, glutathione peroxidase (GPx) and catalase (CAT), remove H_2_O_2_ that is generated either by a two-electron transfer or because of sickling. CAT is usually more significant than GPx because it can convert H_2_O_2_ into H_2_O, without burning cellular-reducing equivalents (glutathione (GSH) or nicotinamide adenine dinucleotide phosphate (NADPH)), which is a more energy-efficient way of eliminating H_2_O_2_. Interestingly, in transgenic sickle mouse models and SCD patients, certain investigations have found reduced CAT activity, but some contradicting reports have indicated instead increased CAT activity in SCD patients [21,23]. However, an increase in CAT activity may represent a defensive mechanism to scavenge H_2_O_2_ [21,23]. Yet, others have found that the activity of CAT in human sickle RBCs is not affected [24]. In disorders such as SCD where vascular oxidative stress is ensued by accumulations of ROS (superoxide anion, hydrogen peroxide, and the hydroxyl radical), vascular oxidative damage has long been linked to exposure to phosphatidylserine on RBCs [21]. Furthermore, in SCD patients, the normal antioxidant capacity of the RBC is impaired due to defects in the availability of antioxidants—lower levels of GSH, and enzymatic antioxidants such as peroxiredoxin 2 and non-enzymatic antioxidants such as vitamins C and E [24]. These findings, when considered collectively, point to oxidative stress playing a significant role in the pathophysiology of SCD [8,21,25].

## 3. Nrf2 Is a Basic Leucine Zipper Transcription Factor That Belongs to the Cap’n’collar Subfamily

**Figure 1:**
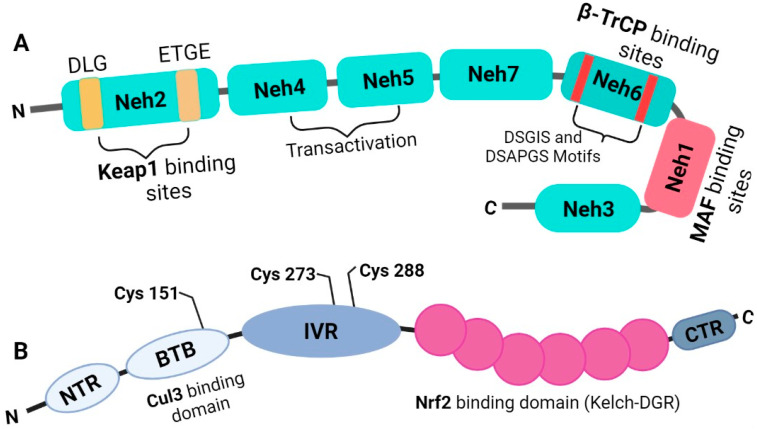
Illustration for Nrf2 and Keap1 Structures: (**A**) Nrf2 is a 589-amino acid long amino acid protein containing seven domains specific for different functions. At C terminal, Neh1 is located between Neh6 and Neh3 domains and has Maf as DNA binding sites. Neh6 has serine-rich DSGIS and DSAPGS motifs, which are degron involved in mediating Nrf2 nuclear degradation. Neh2 domain is located at the N-terminal region that provides the binding sites for Keap1. Similarly, in (**B**) cartoon of Keap1 is illustrated. Kelch-DGR is an Nrf2 binding domain located at the C-terminal region, while Cullin 3 binding domain (BTB) is situated at the N terminal of the protein.

Nrf2 is a cap’n’collar (CNC) transcription factor family member, including the founding member NF-E2p45. The transcription factor NF-E2p45 was initially discovered to bind to the erythroid gene regulatory element NF-E2 that is situated in the promoter region of the heme biosynthetic porphobilinogen deaminase gene (*PBGD*) [26]. Additionally, it was discovered that the transcription factor Nrf2 from humans binds to the gamma-globin gene regulatory region [27]. Along with NF-E2p45, the CNC family also consists of Nrf1, Nrf2, Nrf3, Bach1 (tBTB domain and CNC homolog 1), and Bach2. In contrast to the other proteins in the CNC family, Bach1 and Bach2 act as transcriptional repressors [26]. The basic leucine zipper (bZip) transcription factor is translocated to the nucleus and forms heterodimers with small musculoaponeurotic fibrosarcoma proteins (sMaf) K, G, and F [28]. This heterodimer then recognizes the antioxidant response element (an enhancer sequence) that is present in the regulatory regions of over 250 genes also known as AU-rich elements (AREs) [29]. There are 589 amino acids in Nrf2, containing seven Neh1-7 (N-terminal Nrf2-ECH homology) evolutionarily highly conserved domains. The Neh1 domain is a basic leucine zipper (bZip) motif. This motif recognizes DNA and facilitates its binding, and it is critical for Nrf2 dimerization with sMaf proteins [28]. The Neh2 domain has the ETGE motif located in the hydrophilic loop of the β-loop-β-structure at the C-terminal region and has a strong affinity for the β-propeller of Keap1-DC domain [30]. Similarly, the conserved DLG motif of Neh2 is located in a flexible region upstream of the core β-helix, and also binds Keap1-DC; however, with a noticeably reduced affinity [31]. Neh6, a serine-rich region containing the DSGIS and DSAPGS motifs, works as a degron to mediate Nrf2’s nuclear degradation [32]. The transactivation domains for Nrf2 include Neh4 and Neh5 and serve as the site for HRD1 binding [31,33]. These two domains regulate coordinately the transactivation of different cytoprotective genes [34]. Another domain, Neh7 (209-316 amino acid), was also found in Nrf2 which serves as a binding site for retinoid X receptor α (RXRα) (Figure 1). The direct binding of RXRα to this domain inhibits the functions of Nrf2, and thereby inhibits expression of Nrf2 target genes [35,36].

## 4. Regulation of Nrf2

**Figure 2:**
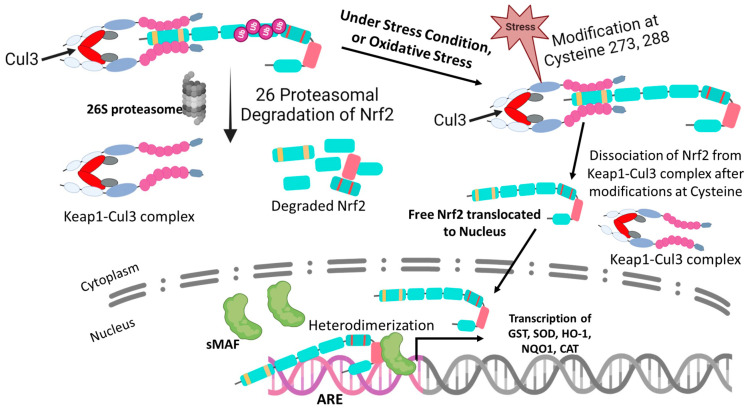
This illustration explains how Keap1–Nrf2 signaling mechanism takes place under both basal and oxidative stress conditions. Under basal conditions, Cul3 and Keap1 heterodimer attaches to Nrf2 and initiates ubiquitination, followed by its 26S proteasomal degradation, which stops nuclear translocation of Nrf2. While under oxidative stress, some of the cysteine residues in IVR domain of the Keap1 are modified that make Keap1 unable to bind with Nrf2. Then, Nrf2 translocates to the nucleus where it heterodimerizes with another protein sMAF and binds to the ARE sequence of various antioxidant enzymes that can mitigate the oxidative burden in the cell.

Regulation of NRF2 expression is mediated via three signaling pathways involving Keap1 (Kelch-like ECH-associated protein 1), HRD1, an E3 ubiquitin ligase involved in protein degradation [37], and E3 ligase adapter β-TrCP (β-transducin repeat-containing protein). These three proteins facilitate NRF2 proteasomal degradation by different mechanisms. As discussed above, Nrf2 has seven highly conserved domains (Neh1–Neh7) that make interactions possible with various different proteins, especially Keap1 [35]. The hydrophilic area of lysine residues (7K) in Neh2 is essential for Keap1-dependent polyubiquitination and Nrf2 degradation, and it also contains the ETGE and DLG motifs that are necessary for the interaction with Keap1 [31,38], whereas, the Keap1 has five domains and 624 amino acid residues. The intervening region (IVR) lies between the BTB and the Kelch domain, two protein–protein interaction motifs. The BTB domain and IVR’s N-terminal region work together to homodimerize Keap1 and connect to Cullin3 (Cul3) [39]. Cul3 is the core protein of the E3 ubiquitin–protein ligase complex, which facilitates target protein ubiquitination and proteasomal breakdown [39]. The interaction with Neh2 is mediated by the C-terminal region and the Kelch domain. With 27 cysteines in human protein, Keap1 has a high concentration of cysteine residues [40,41,42,43]. According to the “hinge and latch” model, the dimer of two keap1 molecules interacts with Nrf2 [44]. Thus, under basal conditions, the Nrf2 protein is tightly controlled by the Keap1–Cul3–E3 ubiquitin ligase complex to keep Nrf2 at its low level. Moreover, another recent research has revealed that the deubiquitinating enzyme USP15 also plays a significant part in controlling the ubiquitination and degradation of Nrf2 [45]. Keap1 is deubiquitinated by USP15, which also stabilizes and improves the E3 ligase activity of the Keap1–Cul3–E3 complex [39,45]. As a result, Nrf2 is eventually degraded. However, under induced conditions or oxidative stress, the controlling activity of the Keap1–Cul3–E3 ubiquitin ligase complex is impaired, and Nrf2 level increased [45]. Keap1 has been hypothesized to release Nrf2 by covalent modifications of the crucial cysteine residues (Cys-151, Cys-273, and Cys-288), since it is a thiol-rich protein and is therefore sensitive to an electrophile [46]. In other words, post-translational modifications in cysteine residues lead to the dissociation of the Cul3-based E3 ligase complex from Keap1. This dissociation helps prevent the proteasomal degradation of Nrf2 and leads to Nrf2 stabilization [47]. After dissociation, Nrf2 accumulates in the cytosol, then translocate to the nucleus, although the de novo generated Nrf2 was also found to accumulate in the cytoplasm and migrate into the nucleus rather than detaching from Keap1 (Figure 2) [48]. In the nucleus, sMaf binds to Nrf2 and forms a heterodimerized form that can recognize and bind to the ARE sequences of various antioxidant enzymes (such as NAD(P)H quinone oxidoreductase 1 (NQO1), glutathione S-transferases (GST), superoxide dismutase (SOD), and heme oxygenase 1 (HO-1), etc.) and promote their expression to cope with oxidative stress [49].

An additional regulatory mechanism via the proteasomal degradation of Nrf2 is mediated by GSK3 (glycogen synthase kinase 3)/β-TrCP [50]. GSK-3α and β-TrCP are serine/threonine protein kinases, members of multiple signaling pathways such as WNT, Hedgehog, and RTK (receptor tyrosine kinase), influencing cellular division, development, and survival [51]. Under normal conditions, these protein kinases are inactive due to phosphorylation by AKT [52]. However, in the active state, GSK-3 phosphorylates the specific serine residue at Neh6 (DSGIS) of Nrf2 and recruits the β-TrCP, which is then tagged for further ubiquitin–proteasome degradation mediated by Cul1/Rbx1 complex [51]. Alternative degradative systems, such as the inositol-requiring enzyme (IRE1), an endoplasmic reticulum (ER)-resident transmembrane protein acting as a proximal sensor of the unfolded protein response, and synoviolin/HRD1, an E3 ubiquitin ligase, are also capable of controlling Nrf2 at the post-transcriptional level [53]. For example, increased endoplasmic reticulum (ER) stress and ROS in liver diseases, can upregulate the XBP1–Hrd1 pathway (an ER stress-response pathway) and downregulate the Nrf2-mediated antioxidant-response pathway [54].

## 5. The Molecular Activation and Cytoprotective Activity of the Keap1-Nrf2 Pathway against Oxidative Stress

Whenever cells encounter an unbalanced redox reaction or high levels of ROS, these cells acquire cytoprotection by activating the Keap1-Nrf2 pathway. This pathway consists of four interlinked constituents: inducers, Keap1 protein (senses the inducers), Nrf2, and target genes that execute the cytoprotection against oxidative stress. Under basal conditions, Keap1 binds to the ETGE and DLG motifs on Nrf2 and brings Nrf2 into the Keap1–Cul3–E3 ubiquitin ligase complex. Subsequently, Nrf2 is being degraded by the Keap1-dependent Cul3–E3 ubiquitin–proteasome pathway [55,56,57]. A cysteine code hypothesis is given, which states that in response to oxidative stress, these 27 cysteines are prime choices for electrophiles and oxidizing agents, because some of them are situated close to basic residues [58]. The functional significance of Cys151, Cys273, and Cys288 has been demonstrated by the fact that Cys151 is necessary for the inducer-induced activation of Nrf2, and that Cys273 and Cys288 are required for its repression [59]. Additionally, Cys226, Cys613, Cys622, and Cys624 residues have recently been found to be important for sensing hydrogen peroxide by forming the disulfide bond in order to maintain the fail-safe mechanism [57]. Although the pattern of modification of these cysteine residues by electrophiles is known as the “cysteine code” [60], the modification of cysteine residues causes conformational changes in the Keap1 that results in the disruption of Nrf2 from Keap1, and therefore inhibits the polyubiquitination of Nrf2 [40,41,43]. Thus, the stabilization of Nrf2 increases its nuclear localization and accumulation. In the nucleus, Nrf2 is heterodimerized with sMaf and then this heterodimerized Nrf2–sMaf complex recognizes the ARE sequences of various antioxidant genes and activates gene transcription [61]. Nrf2, in addition, competes with the transcription factor Bach1 for binding in the ARE motifs of antioxidant genes to regulate cellular oxidative stress levels [62,63].

## 6. Nrf2-Mediated Globin Gene Regulation

**Figure 3:**
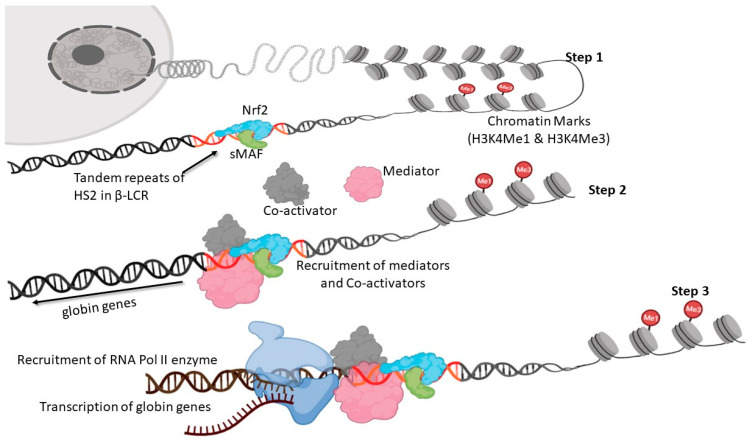
Illustrations show stepwise deciphering of how the heterodimer of Nrf2-sMAF in the nucleus mediates the transcription of globin genes accordingly, after binding at the regulatory sequence of the gene. For globin gene expression, heterodimer of Nrf2 with sMAF binds to the tandem repeats of hypersensitive site 2 (HS2) in β-LCR (β-locus control region). This heterodimer of Nrf2 with sMAF recruits coactivators and mediators to the site and makes the chromatin structure accessible to the RNA polymerase II (Pol II) enzyme. Thereafter, Pol II and general transcription factors (not shown in the illustration) are recruited to transcribe the globin genes.

SCD phenotypic severity can be alleviated by increasing HbF expression to inhibit and thus reduce oxidative stress [64,65,66]. Most studies that propose to develop new SCD therapies aim to reactivate fetal γ-globin expression as their goal [67,68]. Infants with Hb SS have a delay in the fetal γ- to β-globin switch, and HbF levels average 9% at 24 months of age. This observation provided the impetus for widespread research efforts to understand the mechanisms of γ-globin gene regulation to develop strategies to reverse this process in SCD. The efficacy of HbF is due to its ability to dilute HbS levels below the threshold required for polymerization and to influence HbS polymer stability in the sickle RBCs [69]. To increase HbF expression, some research teams have tried to investigate how Nrf2 affects the regulation of the globin genes, particularly gamma globin, although there has not been much research carried out in this area up to now [70,71,72]. The β-locus control region (β-LCR), which has several Dnase1 hypersensitive sites, tightly controls the expression of the globin gene [73]. By interacting with transcription factors that connect these DNA regions to the RNA polymerase machinery, all of these sites are able to exert stimulatory, inhibitory, or more complex activities [74]. Hypersensitive site 2, which has tandem repeats present in the locus control region, helps with the expression of genes present in the globin gene cluster (add reference). These tandem repeats act as binding sites for activating protein 1 (AP1), nuclear factor erythroid 2, and Nrf2 (NF-E2-related factor 2) [27]. A strong acidic activation domain present in Nrf2 may contribute in the transcriptional stimulation of β-globin genes [27], although this function of Nrf2 is still unclear. Therefore, to evaluate the role of Nrf2 in the expression of the globin gene cluster, the human β-globin locus yeast artificial chromosome transgenic/NRF2 knockout (β-YAC/NRF2) mouse model was developed by a research team [75]. NRF2 loss decreased β-globin gene expression during erythropoiesis and eliminated dimethyl fumarate’s ability to increase β-globin transcription [72,76]. In β-YAC/NRF2 mice, it was found that the chromatin marks H3K4Me1 and H3K4Me3 were reduced, and that TATA-binding protein and RNA polymerase II were associated with the promoters of the globin and locus control region (LCR) genes (Figure 3) [75].

## 7. Regulatory Role of Keap1-Nrf2 Heterodimer in Iron, Heme, and Hemoglobin Metabolism

Nrf2 mediates the cytoprotective response against oxidative stress by also influencing the iron-regulatory mechanism. For hemoglobin synthesis, iron is reutilized for RBC production in the spleen, while in an iron-overload condition, liver increases the production of a peptide hormone named hepcidin that maintains iron homeostasis [77]. Hepcidin degrades ferroportin (FPN1), the only iron exporter highly expressed in the basolateral membrane of the small intestine, and, thereby, decreases iron absorption in the small intestine [78,79]. Moreover, macrophages also scavenge heme and hemoglobin and degrade heme into biliverdin, free iron, and carbon monoxide through HO-1 activity [26,80]. This free iron can be stored with ferritin and exported by FPN1 to other cells for further utilization [26,80]. Ferritin is structurally a complex molecule of 24 heavy and light chains (FTH1 and LTH, respectively) in different ratios for various functions. Because FTH1 has oxidase activity, it stores the iron in a stable ferrihydrite form [26,81]. A study has found that the ARE, a 4 kb upstream transcription site in the FTH1 gene, is induced by Nrf2 and is responsible for the expression of FTH1 [81]. Additionally, the available literature suggests that AREs are also located 7 kb upstream of the FPN1 transcription start site and they are implicated in Nrf2-mediated regulation of the FPN1 gene [26,81]. Nrf2 signaling can be suppressed by Bach1, which forms a heterodimer by competing with Nrf2 in binding with the sMaf protein [63]. Heme, an inducer of HO-1, inactivates the Bach1 protein, promoting displacement of Bach1 from the sMaf-occupied HO-1 enhancers; a mechanism followed by Nrf2 binding to these elements [26,63].

## 8. Keap1-Nrf2-Mediated Gamma Globin Chain Regulation in Hemoglobinopathies

To treat SCD and β-thalassemia, HbF induction has shown therapeutic potential [70,72,82]. According to a study, reducing intravascular sickling with HbF has far-reaching effects, including increased nitric oxide (NO) bioavailability and decreased organ oxidative stress [83]. It has been further evidenced that NO can reduce organ oxidative stress, and there is a strong correlation between plasma NO levels and lipid peroxidation [83]. Useful agents in humans, such as hydroxy urea (HU), DNA methyltransferase (DNMT) inhibitors, and butyrate derivatives are less recommended, since they contribute to decreased hematopoiesis and they have long-term negative effects, including DNA mutation and epigenetic alterations [70]. Even though hematopoietic stem cell transplantation and gene therapy are both very successful treatments for hemoglobinopathies, most patients are not receiving either of these treatments due to the high costs, and lack of the necessary high-tech lab facilities. Therefore, more than 50 agents including cancer chemotherapy drugs, DNMT inhibitors, and histone deacetylase inhibitors have been considered to induce gamma globin expression (fetal Hb/HbF) pharmacologically [72,82]. Other studies have shown that HU, butyrate derivatives, and the DNMT inhibitor 5-Aza-2-deoxy-cytidine (decitabine) are also able to induce HbF production in hemoglobinopathy patients [24,66,84]. However, most of them are cytotoxic, damage DNA, alter epigenetic marks on a genome-wide basis, or suppress erythropoiesis [82]. Therefore, the discovery of specific molecules whose function can be affected by small molecules or antibodies is a crucial first step in the development of targeted therapies for hemoglobinopathy patients. Nrf2 through Keap1 directly induces the expression of gamma globin that can compensate for the globin imbalance [70,72], making thus Nrf2 the most plausible target to treat the β-globin hemoglobinopathies. Nrf2 was initially discovered as a DNA binding protein in the β-globin locus, and it was concluded after various reports in which tert-butylhydroquinone (tBHQ), simvastatin, and dimethyl fumarate (DMF) were found to be HbF inducing drugs, by enhancing the gamma globin gene expression through Nrf2 activation in human erythroid progenitors and in SCD mice [72,76,85,86]. More specifically, tBHQ is the substance that promotes Nrf2 nuclear translocation. It is evident that tBHQ can activate Nrf2 in multiple ways, including the degradation of Keap1, which causes Nrf2 to accumulate in the cytosol, by increasing in Nrf2 phosphorylation, and stabilizing Nrf2 ubiquitination, which separates Nrf2 from Keap1 to halts its destruction [70,72].

**Figure 4:**
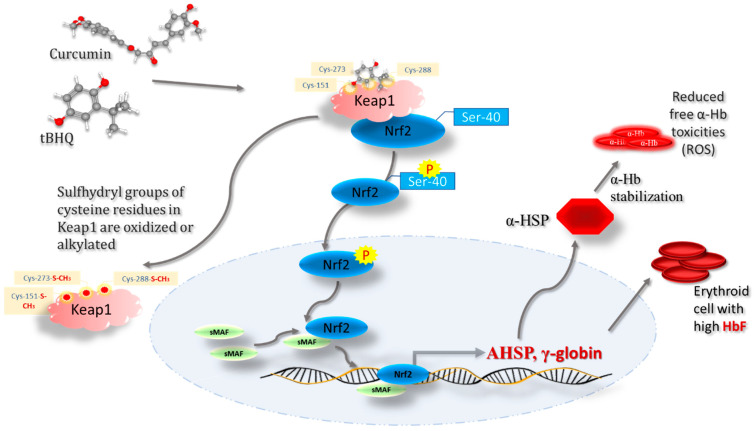
Since the Keap1–Nrf2 interaction is the most crucial part of this signaling, and for Nrf2 to play a cytoprotective role, it must be dissociated from Keap1 in response to oxidative stress. Various (natural and synthetic) molecules involved in Keap1–Nrf2 dissociation have been proposed because of their electrophilic properties. These molecules directly interact with some specific cysteine residues on Keap1; as a result, sulfhydryl groups on cysteines are either oxidized or alkylated (only alkylation is illustrated in the figure), leading to Nef2 dissociation from Keap1. It is also reported that tBHQ/curcumin phosphorylate Nrf2 at Serine-40. Subsequently, Nrf2 translocates to the nucleus, where it heterodimerizes with sMAF and increases the expression of both AHSP and γ-globin. AHSP is involved in the stabilization of free alpha-hemoglobin and helps reduce the free alpha-Hb-generated oxidative stress. On the other hand, the level of HbF is also reported to be high in primary human erythroid cells due to higher expression of γ-globin gene.

Reducing the amount of Keap1 causes a release of Nrf2 in the cytosol, then Nrf2 translocate to the nucleus where it ultimately controls ROS levels by inducing the expression of the AHSP (alpha hemoglobin stabilizing protein) gene in response to increased oxidative stress due to the accumulation of alpha globin chains [87,88]. Interestingly, AHSP expression has been found to be elevated among sickle cell disease patients under HU therapy [88]. This suggests that AHSP also plays a role in reducing oxidative stress. Therefore, it is likely that the combined effects of multiple Nrf2–Keap1 associated signaling pathways are responsible for the protective effects in β-globinopathies as well [89]. This hypothesis was supported by another study showing that using a Keap1 modulator increases nuclear Nrf2 levels, where Nrf2 modulates the expression of various genes contributing to the enhanced expression of the gamma globin gene (Figure 4) [90]. Chromatin immunoprecipitation (ChIP) analysis in the Keap1 knock-out cells demonstrated the recruitment of Nrf2 in the region of ARE of the promoter sequences of both NAD(P)H quinone dehydrogenase 1 (NQO1) and γ-globin genes [90].

## 9. Keap1–Nrf2 Signaling as a Potential Therapeutic Target in SCD

As per the paragraph, it is now clear that an enhanced expression of the gamma globin gene substantially ameliorates the complications of SCD. Additionally, the dissociation of Nrf2 from Keap1 substantially activates the expression of numbers of antioxidant enzymes in SCDs, and helps to maintain the balance between metabolic redox reactions [91]. Therefore, Keap1–Nrf2 signaling should also be considered as one of the plausible therapeutic target sites for the management of SCD. Here, there are some other approaches that have been found to be therapeutically significant to reduce the pathophysiology of SCD.

Aforesaid, activated Nrf2 further activates GSH and HO-1 along with various antioxidant enzymes [49]. Reduced GSH is tripeptide of L-glutamate, cysteine, and glycine synthesized by the reactions catalyzed by both gamma-glutamyl cysteine ligase and GSH synthetase in the cytosol [92,93]. GSH is actively oxidized by ROS and reduced to GSSG (glutathione disulfide), thus, providing cytoprotection [92,93]. It has been observed that GSH and glutamine levels are very much reduced in SCD, and supplementation of glutamine ameliorates the redox imbalance, pain crises, and other complications of the SCD [94,95]. Hence, it can be hypothesized that the Keap1-Nrf2 signaling pathway is potentially involved in regulating cellular GSH in SCD [96]. Similar results were observed when Keap1 was mutated or cells were under oxidative stress, showing that Nrf2 is active, but these cells were deficient in glutamine because of the increase in the activity of the cystine-glutamate antiporter protein xCT [97,98]. The xCT is encoded by SLC7A11, and activated by Nrf2, facilitating cystine entry into the cell [97,98]. The elevated level of xCT reduces the anaplerosis of the tricarboxylic acid (TCA) cycle and makes the cells dependent on glutamine catabolism to glutamate to support xCT flux [98]. That is why the supplementation of glutamine helps ameliorate SCD complications [94,99]. Similarly, Nrf2 encourages the usage of cysteine in the biosynthesis of the antioxidant GSH for the purpose of ROS detoxification [100,101]. Additionally, Nrf2 boosts the activity of gamma-glutamyl–cysteine ligase (GCL), a heterodimeric enzyme composed of both GCL catalytic subunit (GCLC) and GCL modifier subunit (GCLM), which catalyzes the first stage of GSH biosynthesis [101]. GCL makes it easier for cysteine and glutamate to combine and form gamma-glutamyl cysteine, which is a precursor to GSH [56,99,100,101]. Moreover, a group of scientists in 1997 observed the dynamic properties of S-nitrosohemoglobin in vasodilation control [102]. They have reported that the thiol groups presented in cysteine residues of beta-globin exhibit inhibitory effects of NO, due to the reaction between NO and reduced sulfhydryl groups (−SH) that generates S-nitrosothiols (RSNOs) [102,103]. Thiols such as GSH that possess a high affinity towards NO take part in trans-nitrosylation processes, where nitroso-Hb (SNO-Hb) transfers NO to the thiol to create nitrosoglutathione (GSNO) with good vasodilatory effects and leading to reduced pain crises in SCD [102].

**Figure 5:**
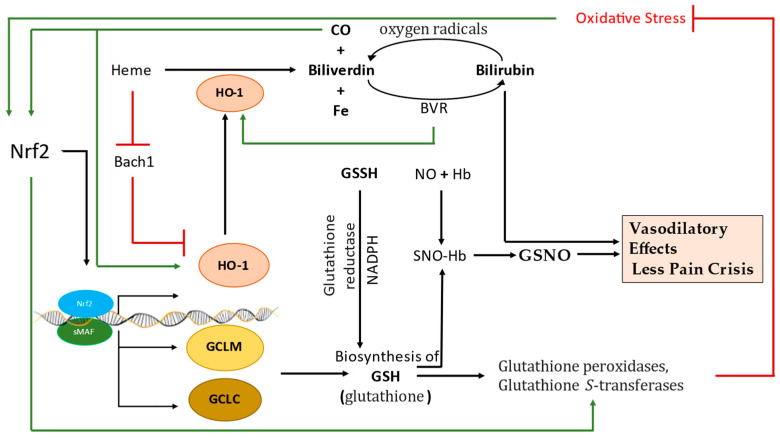
Flow diagram depicting the complete pathway indicating how Nrf2 directly or indirectly ameliorates SCD complications through HO-1 and GSH. HO-1 is a crucial enzyme involved in heme catabolism and cleaves heme into CO, Fe cations and biliverdin. CO positively induces Nrf2 and HO-1 expression, which further help to maintain oxidative balance. On the other hand, biliverdin converts to its reduced form called bilirubin by biliverdin reductase (BVR). Bilirubin the vasodilator byproduct can reduce pain crises. Similarly, Nrf2 maintains the biosynthesis of GSH, a substrate for the antioxidant enzymes GPx and GST. GSH also has an affinity towards NO and pulls the NO from nitroso-Hb to form GSNO. GSNO has been studied as the one of the strongest naturally occurring vasodilatory agents.

Further, Belcher et al., observed the beneficial effects of carbon monoxide (CO) in reducing sickling of erythrocytes in a mouse model of SCD [104]. Inhaling CO also reduced vascular stasis in animal models of SCD [104,105,106]. CO was, in addition, reported to be directly involved in anti-inflammatory and antioxidant actions along with the stimulation of heme oxygenase-1 (HO-1) enzyme in SCD [104]. HO-1 is an antioxidant and anti-inflammatory enzyme primarily involved in the catalytic cleavage of heme and its conversion into biliverdin, CO and iron cations [104]. Along with preventing HbS polymerization and sickling, and vaso-occlusion, CO is thought to play a role in enhancing the expression and activity of HO-1 as a positive feedback protective mechanism against hypoxia as well. CO can cause increases in nuclear Nrf2, which promotes HO-1’s expression and activity, together with decreases in NF-ĸB activation and the expression of adhesion molecules such as P-selectin and von Willebrand factor (VWF) [104,107]. In addition to CO, biliverdin produced by HO-1 converts into bilirubin by biliverdin reductase (BVR). Bilirubin has vasodilating properties. Bilirubin can also reduce oxygen species by scavenging the reactive oxygen. Bilirubin, when reacting with oxygen species, is converted back to biliverdin [108]. The HO-1-derived biliverdin lessens the angiotensin II-mediated vascular damage by inhibiting the activities of NADPH oxidases and protein kinase C (PKC) [109]. We therefore hypothesize that Nrf2, by inducing HO-1, may also have vasodilatory effects via biliverdin (Figure 5). To additionally evaluate the role Nrf2 in SCD, scientists have recently observed reduced inflammation, and mRNA expression of the proinflammatory cytokines, tumor necrosis factor (TNF)α and interleukin (IL)1β, using the Cre-recombinase system [110]. They found that Nrf2 activation in monocytes/granulocytes, and endothelial cells ameliorates the pathophysiology of SCD, suggesting that targeting Nrf2 activation could be a plausible therapeutic approach to ameliorate SCD pathology [110]. We also suggest that MiRNA-based research may further support the notion of targeting Nrf2 therapeutically for the management of SCD severity. It has been identified that miRNAs (miR-153, miR-27a, miR-142-5p, miR-144, miR-28, and miR-34a) are involved in the reduced expression and activity of Nrf2 in various diseases [111]. Similarly, miR-144 was evidenced to be highly expressed among the most severe phenotypes in SCD patients [112].

## 10. Keap1-Nrf2 Being Targeted Therapeutically in Various Diseases

Nrf2 affects GSH, thioredoxin, HO-1, and NQO1, but this Nrf2 pathway can also be activated by DMF to protect mitochondria from damage caused by ROS [113]. Similarly, a mouse ovary-based study has suggested that the DMF-induced Keap1-Nrf2 pathway can improve age-associated infertility [71]. Due to high oxidative stress, mitochondrial biogenesis is impaired in multiple sclerosis (MS) patients [113,114], suggesting that it may have implications for MS pathophysiology and therapy. The use of DMF can also oxidize sulfhydryl (-SH) groups of Keap1, eventually activating Nrf2, subsequently inducing mitochondrial biogenesis and gene expression [113,114]. Chalcones have also been reported to trigger cytoprotective proteins, upregulate multidrug resistance-associated proteins, stimulate the Nrf2 signaling pathway, and increase the expression of antioxidant genes that are Nrf2 regulated [115]. Due to their soft electrophilicity, chalcones are less likely to have harmful off-target effects, and they are also unlikely to cause mutagenicity and carcinogenicity. Additionally, due to their low toxicity, structural variety, structural rearrangement capability, and the existence of an unsaturated carbonyl group, chalcones make good candidates for drugs that target Nrf2-dependent pathologies [115]. Similarly, sulforaphane and curcumin are the classical Nrf2 activators that affect the expression of aldo-keto reductase (AR), NQO1, GST, and HO-1, and lead ultimately to the activation of a xenobiotic response in the cells [116,117]. Curcumin may indirectly phosphorylate Nrf2 at serine and/or threonine-rich regions and facilitate the nuclear transition of Nrf2 (Figure 3) [117]. In addition, sulforaphane can directly interact with sensor cysteine thiol(s) of Keap1 and diminish its inhibitory effect on Nrf2 [116,117]. Acacetin, a flavonoid, is another drug with antioxidative effects mediated by the phosphorylation of Nrf2 at Ser40 and the inhibition of Keap1 expression via the MsrA-Nrf2/Keap1 pathway [118]. Recent reports have also found esculetin to be a potent modulator of Nrf2 expression and downregulator of 2,2-diphenyl-1-picrylhydrazyl (DPPH) radical generation in particular cells (Table 1) [119]. Medicinal and therapeutic properties of various natural and synthetic small molecules including sulforaphane, curcumin, oltipraz and bardoxolone methyl have been investigated and found to be significantly involved in the expression of ARE-dependent cytoprotective genes [116,117,120]. However, the majority of these Nrf2-activating molecules are electrophilic species or metabolically transformed electrophiles that interact with sulfhydryl groups of cysteine residues in Keap1, by either oxidation or alkylation, hence called indirect inhibitors of Keap1–Nrf2 interaction (Figure 4). Thus, direct inhibition of the Keap1–Nrf2 protein–protein interaction is now the most plausible approach to activate the Nrf2 and its downstream signaling [121].

## 11. Conclusions

The cyclic polymerization and depolymerization of HbS are the hallmark of SCD, causing oxidative stress. This oxidative stress further increases the SCD-associated complications especially severe anemia and recurrent painful vaso-occlusive crises, due respectively to increased hemolysis and sickle blood-cell adhesion to the vascular endothelium and to adherent leukocytes. Hence, to maintain the redox homeostasis of the sickle erythrocyte, ROS are counterbalanced by numerous intricate antioxidant systems, among which the Nrf2/Keap1 signaling is the most prominent one. Moreover, Nrf2 may enhance gamma-globin gene expression since it has a binding affinity towards the β-locus control region. In this review, it has been concluded that a high level of HbF has cytoprotective roles in SCD. β-locus control region (β-LCR) has some tandem repeats in the HS-2 site that provide a binding site to the Nrf2 gene. Therefore, Nrf2-Keap1 signaling can be a plausible target for enhancing gamma globin expression. However, the exact function of Nrf2 on gamma globin gene expression requires further extensive investigative research.

## Figures and Tables

**Table 1:** NRF2-Keap1 modulating drugs, targeted pathways and their mode of action in response to oxidative stress.

Drug/Chemical/Agents	Targeted Pathway	Disease(s)/Complications	Mode of Action	Ref.
Esculetin	A potent inhibitory effect on NO, iNOS and DPPH radicals via modulating Nrf2	Inflammation	Esculetin substantially suppressed NF-B p65 nuclear translocation at a higher concentration of 20 M. Esculetin boosted Nrf2 expression while reducing DPPH radical production in macrophage cells at the same high concentration.	[119]
Dimethyl fumarate (DMF)	Antioxidant NRF2 transcriptional pathway and mitochondrial biogenesis	Multiple sclerosis (MS)	DMF can oxidize Keap1’s sulfhydryl (-SH) groups, which activates Nrf2 and causes mitochondrial biogenesis and the activation of many genes.	[113,114,122]
Acacetin	MsrA-Nrf2/Keap1 pathway	Atherosclerosis	Acacetin’s antioxidative effects are mediated by phosphorylation of Nrf2 at Ser40 and inhibition of Keap1 expression via the MsrANrf2/Keap1 pathway.	[118]
Wogonin	Activated Nrf2 signaling, and inhibited NF-κB-regulated pro-inflammatory signaling	Sepsis or septic liver injury	By activating Nrf2, wogonin encourages the production of antioxidative enzymes such NQO1, GST, HO1, SOD1 and SOD2 in hepatocytes. Additionally, wogonin-induced Nrf2 activation prevented the production of pro-inflammatory cytokines under NF-κB control.	[123]
Isosalipurposide (ISPP)	Keap1-Nrf2 signaling	Oxidative injury of hepatocytes	ISPP causes ERK and AMPK to be phosphorylated along with an increase in Nrf2 phosphorylation.	[124]
tBHQ	Keap1-Nrf2 signaling	β-thalassemia and sickle cell disease	Both improved nuclear localization of Nrf2 and boosted expression of a large panel of Nrf2 dependent genes, tert-butyl hydroquinone (tBHQ) provided greater protection against oxidative stress.	[70]
Curcumin	Keap1-Nrf2 Signaling and Akt/Nrf2 pathway	Neuroprotection against oxidative stress; cancer chemopreventive agent sulforaphane	It facilitates Nrf2’s nuclear translocation by phosphorylating it at serine-40 and/or threonine-rich areas.	[116,117]
Resveratrol	Keap1-Nrf2 Signaling	Vasoprotection in animal models of type 2 diabetes and aging	It demonstrates electrophilic properties and interacts with Keap1’s cysteine residues (Cys151, Cys257, Cys273, Cys288, and Cys297) via oxidation or alkylation to remove Nrf2 from Keap1.	[47,125]
RTA 408 (Omaveloxolone)	Keap1-Nrf2 Signaling	Diabetic wounds, Friedreich’s ataxia, ocular inflammation	RTA-408 promotes Nrf2-mediated antioxidant activity	[126,127]
Ursodiol (ursodeoxycholic acid) or UDCA	Keap1-Nrf2 signaling	Cholestatic liver diseases	The efflux transporters, detoxifying enzymes such as NQO-1, and antioxidative stress genes such as γ-GCS are substantially increased in the liver by UDCA-induced Nrf2 activation.	[128]
CXA-10 (10-nitro-9(E)-octadec-9-enoic acid	Keap1-Nrf2 signaling	Chronic kidney disease (CKD)	It alters Keap1’s essential cysteine residues (Cys273 and 288) and aids in the release of Nrf2, which activates the ARE and upregulates the synthesis of antioxidant and detoxifying proteins.	[129]
